# Efficacy and safety of darolutamide in Japanese patients with nonmetastatic castration-resistant prostate cancer: a sub-group analysis of the phase III ARAMIS trial

**DOI:** 10.1007/s10147-020-01824-5

**Published:** 2020-11-23

**Authors:** Hiroji Uemura, Hisashi Matsushima, Kazuki Kobayashi, Hiroya Mizusawa, Hiroaki Nishimatsu, Karim Fizazi, Matthew Smith, Neal Shore, Teuvo Tammela, Ken-ichi Tabata, Nobuaki Matsubara, Masahiro Iinuma, Hirotsugu Uemura, Mototsugu Oya, Tetsuo Momma, Mutsushi Kawakita, Satoshi Fukasawa, Tadahiro Kobayashi, Iris Kuss, Marie-Aude Le Berre, Amir Snapir, Toni Sarapohja, Kazuhiro Suzuki

**Affiliations:** 1 Department of Urology and Renal Transplantation, Yokohama City University Medical Center, 4-57 Urafune-cho, Minami-ku, Yokohama, 232-0024 Japan; 2 Department of Urology, Tokyo Metropolitan Police Hospital, 4-22-1 Nakano, Nakano-ku, 164-8541 Japan; 3 Department of Urology, Yokosuka Kyosai Hospital, 1-16 Yonegahamadori, Yokosuka, 238-8558 Japan; 4 Department of Urology, National Hospital Organization, Shinshu Ueda Medical Center, 1-27-21 Midorigaoka, Ueda, 386-8610 Japan; 5 Department of Urology, The Fraternity Memorial Hospital, 2-1-11 Yokoami, Sumida-ku, 130-8587 Japan; 6 Institut Gustave Roussy, 39 Rue Camille Desmoulins, 94805 Villejuif Cedex, France; 7 Massachusetts General Hospital Cancer Center, 55 Fruit Street, Boston, MA 02114 USA; 8 Carolina Urologic Research Center, 823 82nd Parkway, Myrtle Beach, SC 29572 USA; 9 Tampere University Hospital, Urologian poliklinikka, PL 2000, Teiskontie 35, 33521 Tampere, Finland; 10 Department of Urology, Kitasato University Hospital, 1-15-1 Kitazato Minami-ku, Sagamihara, 252-0375 Japan; 11 Department of Breast and Medical Oncology, National Cancer Center Hospital East, 6-5-1 Kashiwanoha, Kashiwa, 277-8577 Japan; 12 Department of Urology, National Hospital Organization, Mito Medical Center, 280 Sakuranosato Ibarakimachi, Higashiibaraki, 311-3193 Japan; 13 Department of Urology, Kindai University, 377-2, Onohigashi, Osakasayama, 589-8511 Japan; 14 Department of Urology, Keio University, 35 Shinano-machi, Shinjuku-ku, 160-8582 Japan; 15 Department of Urology, National Hospital Organization, Saitama National Hospital, 2-1 Suwa, Wako, 351-0102 Japan; 16 Department of Urology, Kobe City Medical Center General Hospital, 2-1-1 Minatojimaminamimachi Chuo-ku, Kobe, 650-0047 Japan; 17 Division of Urology, Chiba Cancer Center, 666-2, Nitona-cho, Chuo-ku, Chiba, 260-8717 Japan; 18 Department of Urology, Fukui Prefectural Hospital, 2-8-1 Yotsui, Fukui, 910-8526 Japan; 19 Clinical Statistics, Bayer AG, Building P300, B342, 13342 Berlin, Germany; 20 Bayer Healthcare SAS, 220 Avenue de la Recherche, 59120 Loos, France; 21 Orion Corporation Orion Pharma, Orionintie 1, P.O. Box 65, FI-02101 Espoo, Finland; 22 Department of Urology, Gunma University, 3-39-15 Showa-machi, Maebashi, 371-8511 Japan; 23 Present Address: PCI Biotech, Ullernchausseen 64, 0379 Oslo, Norway

**Keywords:** Nonmetastatic castration-resistant prostate cancer, Androgen receptor inhibitor, Metastasis-free survival, Efficacy, Safety, Japanese

## Abstract

**Background:**

Darolutamide, an oral androgen receptor inhibitor, has been approved for treating nonmetastatic castration-resistant prostate cancer (nmCRPC), based on significant improvements in metastasis-free survival (MFS) in the ARAMIS clinical trial. Efficacy and safety of darolutamide in Japanese patients are reported here.

**Methods:**

In this randomized, double-blind, placebo-controlled phase III trial, 1509 patients with nmCRPC and prostate-specific antigen (PSA) doubling time ≤ 10 months were randomized 2:1 to darolutamide 600 mg twice daily or matched placebo while continuing androgen deprivation therapy. The primary endpoint was MFS.

**Results:**

In Japan, 95 patients were enrolled and randomized to darolutamide (*n* = 62) or placebo (*n* = 33). At the primary analysis (cut-off date: September 3, 2018), after 20 primary end-point events had occurred, median MFS was not reached with darolutamide vs. 18.2 months with placebo (HR 0.28, 95% CI 0.11–0.70). Median OS was not reached due to limited numbers of events in both groups but favored darolutamide in the Japanese subgroup. Time to pain progression, time to PSA progression, and PSA response also favored darolutamide. Among Japanese patients randomized to darolutamide vs. placebo, incidences of treatment-emergent adverse events (TEAEs) were 85.5 vs. 63.6%, and incidences of treatment discontinuation due to TEAEs were 8.1 vs. 6.1%.

**Conclusions:**

Efficacy outcomes favored darolutamide in Japanese patients with nmCRPC, supporting the clinical benefit of darolutamide in this patient population. Darolutamide was well tolerated; however, due to the small sample size, it is impossible to conclude with certainty whether differences in the safety profile exist between Japanese and overall ARAMIS populations.

**Electronic supplementary material:**

The online version of this article (10.1007/s10147-020-01824-5) contains supplementary material, which is available to authorized users.

## Introduction

Globally, prostate cancer poses a major health issue among men. In 2018, there were an estimated 1,276,106 new diagnoses of, and 358,989 new deaths from, prostate cancer worldwide [1]. In Japan, prostate cancer ranked as the fourth most common cancer in men in 2014 [2]. In 2018, there were an estimated 70,654 new cases of prostate cancer and 12,424 deaths from prostate cancer [3].

Prostate cancer progression is mediated by the androgen receptor signaling pathway [4], and androgen deprivation therapy (ADT) is considered the standard of care for many patients with recurrent disease [5]. Most patients eventually develop resistance to ADT and progress to castration-resistant prostate cancer (CRPC) [6], which is characterized by rising levels of prostate-specific antigen (PSA) despite castrate levels of testosterone [7]. CRPC in the absence of detectable metastases with conventional imaging is classified as nonmetastatic CRPC (nmCRPC) [7]. Of 249,053 Japanese patients with prostate cancer included in the large-scale, Medical Data Vision health claims database, 1236 cases were identified as nmCRPC between 2003 and 2018, although limitations of claims databases may underestimate this proportion (0.50%) [8]. An earlier analysis of population cancer registries from 28 countries reported the 5-year prevalence of nmCRPC in Asia (including Japan, China, India, Russia, and Turkey) as 3% in 2013 [9].

Patients with nmCRPC are at substantial risk of progressing to metastatic CRPC (mCRPC) [5]. In a 2005 study by Smith et al., 33% of patients with nmCRPC developed bone metastases within 2 years of follow-up [10]. Metastatic disease carries a poor prognosis, with shorter overall survival (OS) compared to patients without metastases [11]. Given that the nmCRPC population is largely asymptomatic [12], delaying the development of metastasis and hence maintaining the quality of life in these patients is a key therapeutic goal, especially when treatment beyond ADT may be required.

Apalutamide and enzalutamide are androgen receptor inhibitors approved for the treatment of nmCRPC in combination with ADT [13–18]. There were specific treatment-related adverse effects more frequently reported with these agents than with placebo, including fatigue, cognitive impairment, seizures, falls, and fractures. In addition, rash and hypothyroidism were observed with apalutamide, while hypertension and major adverse cardiovascular events were observed with enzalutamide [19, 20].

Darolutamide is a structurally distinct androgen receptor inhibitor [21] approved in combination with ADT for treating men with nmCRPC [22–25], after demonstrating significantly prolonged metastasis-free survival (MFS) in the primary analysis of the phase III ARAMIS trial (median 40.4 months vs. 18.4 months; hazard ratio (HR) 0.41, 95% confidence interval (CI) 0.34–0.50, *P* < 0.001; data cut-off September 3, 2018) [26]. Darolutamide also significantly improved OS (HR 0.69, 95% CI 0.53–0.88, *P* = 0.003) at the final analysis (data cut-off November 15, 2019) [27]. Darolutamide exhibits a favorable safety profile: at the primary analysis, incidences of treatment-emergent adverse events (TEAEs) ≥ 5% were generally similar between darolutamide and placebo groups [26]. Furthermore, the incidence of key adverse events (AEs) known to be associated with second-generation androgen receptor inhibitors, including falls, hypertension, and central nervous system (CNS)-related effects, was similar between treatment groups at the primary and final analyses [26]. The low blood–brain barrier penetration of darolutamide, as observed in rodent models and supported by a neuroimaging study in humans [28, 29], may be associated with a low risk of CNS adverse effects. In addition, patient quality of life was maintained with darolutamide treatment [26, 30], and the risk of clinically relevant drug–drug interactions between darolutamide and comedications used in men with nmCRPC was low [31]. Phase I/II studies of mCRPC also support the tolerability of darolutamide and its low risk of TEAEs [32–34] and no differences were observed in safety and pharmacokinetics in Japanese patients relative to Western patients in a small cohort (*n* = 9) [35].

Here, we evaluated the efficacy and safety of darolutamide in Japanese patients with nmCRPC from pre-planned subgroup analyses of the primary data from the ARAMIS trial (data cut-off September 3, 2018).

## Patients and methods

### Study design and patients

The design and patient eligibility of this randomized, double-blind, placebo-controlled, phase III trial (NCT02200614) have been reported previously [26]. Briefly, patients aged ≥ 18 years had histologically or cytologically confirmed adenocarcinoma of the prostate, CRPC, a baseline PSA level of ≥ 2 ng/mL, a PSA doubling time (PSADT) of ≤ 10 months, and an Eastern Cooperative Oncology Group (ECOG) performance status of 0 or 1. Patients were excluded if they had a history of metastatic disease or distant metastases detected by whole body radionuclide bone scan and computed tomography (CT), or magnetic resonance imaging (MRI) of the pelvis, abdomen and chest; presence of pelvic lymph nodes < 2 cm in the short axis below the aortic bifurcation was allowed. Patients with prior seizures or conditions predisposing to seizure were permitted to enter this trial.

The review board at each participating institution approved the trial, which was conducted in compliance with the principles of the Declaration of Helsinki and in accordance with the International Conference on Harmonisation Guidelines for Good Clinical Practice. An independent Data and Safety Monitoring Board reviewed unblinded safety data throughout the trial. All patients provided written informed consent.

### Randomization and treatment

Patients were randomized 2:1 to receive darolutamide 600 mg (two tablets of 300 mg) twice daily with food or matched placebo in a double-blind manner. Randomization was stratified by PSADT (≤ 6 vs. > 6 months) and the use of osteoclast-targeted therapy at randomization (yes vs. no). Patients continued treatment until protocol-defined progression, intolerable AEs, or withdrawal of consent. Patients continued receiving ADT (luteinizing hormone-releasing hormone agonist or antagonist) throughout the trial. Patients who initiated a prohibited therapy before confirmation of metastasis were required to discontinue study treatment, and thereafter, were followed for survival status [26].

### Assessments

Patient demographics, relevant medical history, and other pertinent clinical conditions were recorded at screening. Vital signs and blood samples for laboratory safety assessments were obtained at the study research center at screening and every scheduled visit (Day 1, Day 15, Day 29, at 16 weeks, and at 16-week intervals thereafter). Serum PSA concentrations and pain [evaluated with the Brief Pain Inventory Short Form (BPI-SF) questionnaire] were assessed at screening, Day 1, Week 16, and at every subsequent visit until the end of study or death. Disease assessments, including evaluation of ECOG performance status, chest, abdomen, and pelvic CT/MRI, and ^99m^Tc bone scintigraphy were performed at screening, Week 16, and at every subsequent 16-week visit. All imaging was evaluated both locally and by blinded independent central review.

Data on TEAEs, including type, severity (according to the National Cancer Institute Common Terminology Criteria for Adverse Events, version 4.03), seriousness, and whether they were related to the study treatment according to investigator assessment, were recorded at each visit.

### Endpoints

The primary endpoint was MFS, defined as the time from randomization to confirmed evidence of distant metastasis on imaging or death from any cause, whichever occurred first. Secondary endpoints comprised OS, time to pain progression (defined as an increase of ≥ 2 points from baseline as assessed using the BPI-SF questionnaire or start of opioid treatment for cancer pain, whichever occurred first), time to first cytotoxic chemotherapy, and time to first symptomatic skeletal event (SSE; defined as external beam radiation therapy to relieve skeletal symptoms, new symptomatic pathologic bone fracture, the occurrence of spinal cord compression, or tumor-related orthopedic surgical intervention). Exploratory endpoints included time to PSA progression [defined according to Prostate Cancer Working Group 2 (PCWG2) criteria [36]] and PSA response [defined as the percentage of patients who experienced a decline from baseline in the PSA level of ≥ 50% (according to PCWG2 criteria [36]) or ≥ 90%].

### Statistical analysis

Statistical analysis of efficacy and safety endpoints in the Japanese patient population was conducted as previously described for the global population [26]. Briefly, MFS, all secondary endpoints, and time to PSA progression were analyzed using a stratified log-rank test. Kaplan–Meier curves, including median survival times and their 95% CI were calculated; the HR was calculated with a Cox proportional-hazards model. The percent of patients with a PSA response was analyzed using the Cochran–Mantel–Haenszel test.

Statistical analysis and subject data listings were performed with SAS^®^ for UNIX (SAS Institute Inc., Cary, NC, USA). Incomplete event occurrence dates were imputed as the earliest possible date.

Efficacy was evaluated in the intention-to-treat population, which comprised all randomized patients. Safety was evaluated in the safety population, which comprised all randomized patients who received at least one dose of any study drug.

## Results

### Patients

In total, 95 Japanese patients from the ARAMIS overall population (*N* = 1509) were randomized to darolutamide (*n* = 62) or placebo (*n* = 33). The clinical cut-off date for the primary analysis was September 3, 2018. In this subset of Japanese patients, baseline demographics and clinical characteristics were well balanced between treatment groups except for age, primary tumor classification, and use of osteoclast-targeted therapy. More patients were aged ≥ 85 years (19.4 vs. 6.1%) or had T3a disease (40.3 vs. 18.2%), fewer patients used osteoclast-targeted therapy (8.1 vs. 15.2%), and the median time from first prior ADT was longer (64.8 vs. 53.6 months) in the darolutamide group than in the placebo group (Table 1).

**Table 1:** Baseline demographics and clinical characteristics

Characteristic	Japanese subgroup		Overall ARAMIS population	
	Darolutamide (*N* = 62)	Placebo (*N* = 33)	Darolutamide (*N* = 955)	Placebo (*N* = 554)
Median age (range), years	77.0 (56–90)	76.0 (56–87)	74.0 (48–95)	74.0 (50–92)
Age group (years), *n* (%)				
< 65	3 (4.8)	3 (9.1)	113 (11.8)	84 (15.2)
65–74	20 (32.3)	10 (30.3)	373 (39.1)	216 (39.0)
75–84	27 (43.5)	18 (54.5)	384 (40.2)	209 (37.7)
≥ 85	12 (19.4)	2 (6.1)	85 (8.9)	45 (8.1)
Median time from initial diagnosis (range), months	71.1 (11.9–230.9)	82.6 (15.2–206.3)	86.2 (2.6–337.5)	84.2 (0.5–344.7)
Presence of lymph nodes by central imaging review, *n* (%)				
Yes	10 (16.1)	6 (18.2)	100 (10.5)	66 (11.9)
No	52 (83.9)	27 (81.8)	855 (89.5)	488 (88.1)
Primary tumor classification, *n* (%)				
T2: tumor confined within the prostate	1 (1.6)	1 (3.0)	110 (11.5)	58 (10.5)
T3a: unilateral or bilateral extracapsular extension	25 (40.3)	6 (18.2)	113 (11.8)	49 (8.8)
Gleason score, *n* (%)				
< 7	4 (6.5)	2 (6.1)	217 (22.7)	142 (25.6)
≥ 7	58 (93.5)	30 (90.9)	711 (74.5)	395 (71.3)
Median serum PSA (range), ng/mL	4.5 (1.9–66.1)	4.2 (1.8–99.5)	9.0 (0.3–858.3)	9.7 (1.5–885.2)
PSA doubling time				
Median (range), months	4.2 (1.2–9.4)	4.4 (1.4–7.1)	4.4 (0.7–11.0)	4.7 (0.7–13.2)
≤ 6 months, *n* (%)	51 (82.3)	24 (72.7)	667 (69.8)	371 (67.0)
> 6 months, *n* (%)	11 (17.7)	9 (27.3)	288 (30.2)	183 (33.0)
Median serum testosterone (range), nmol/L	0.6 (0.3–1.4)	0.6 (0.2–1.4)	0.6 (0.2–25.9)	0.6 (0.2–7.3)
ECOG performance status, *n* (%)				
0	55 (88.7)	31 (93.9)	650 (68.1)	391 (70.6)
1	7 (11.3)	2 (6.1)	305 (31.9)	163 (29.4)
Use of osteoclast-targeted therapy, *n* (%)				
Yes	5 (8.1)	5 (15.2)	31 (3.2)	32 (5.8)
No	57 (91.9)	28 (84.8)	924 (96.8)	522 (94.2)
Prior hormonal therapy, *n* (%)				
1	1 (1.6)	1 (3.0)	177 (18.5)	103 (18.6)
≥ 2	61 (98.4)	32 (97.0)	727 (76.1)	420 (75.8)
Not applicable^a^	0	0	51 (5.3)	31 (5.6)
Primary treatment, *n* (%)				
Hormonal therapy	40 (64.5)	19 (57.6)	403 (42.2)	252 (45.5)
Orchiectomy	1 (1.6)	0	91 (9.5)	50 (9.0)
Prostatectomy	8 (12.9)	8 (24.2)	239 (25.0)	134 (24.2)
Radiotherapy	3 (4.8)	5 (15.2)	177 (18.5)	89 (16.1)
Active surveillance	2 (3.2)	0	12 (1.3)	7 (1.3)
Other	8 (12.9)	1 (3.0)	32 (3.4)	22 (4.0)
Median time from first prior ADT (range), months^b^	64.8 (9.9–235.0)	53.6 (14.8–206.8)	64.2 (0.9–644.4)	61.9 (2.9–386.6)

*ADT* androgen deprivation therapy, *ECOG* Eastern Cooperative Oncology Group, *PSA* prostate-specific antigen

^a^Patients who underwent orchiectomy were not required to have been receiving hormonal drug therapy

^b^ADT defined as luteinizing hormone-releasing hormone agonist/antagonists, orchiectomy, or antiandrogens (androgen-receptor inhibitors)

The Japanese patient population showed some variation in baseline disease characteristics from the overall global ARAMIS population. Japanese patients tended to be older and had a lower median baseline PSA than the overall ARAMIS population. Compared to the overall ARAMIS population, more Japanese patients had a PSADT ≤ 6 months, ECOG performance status 0, T3a disease, Gleason score ≥ 7, and had received ≥ 2 prior hormonal therapies (Table 1).

The median duration of treatment was 14.8 months (range 0.4–26.1 months) with darolutamide and 10.9 months (range 2.1–18.6 months) with placebo in the Japanese subgroup (Supplementary Table 1). In this study, 93.5% of patients with darolutamide and 93.9% of patients with placebo received ≥ 90% of the planned dose (Supplementary Table 2). Treatment duration and dosage administered were comparable between Japanese and overall ARAMIS populations.

## Efficacy

**Fig. 1:**
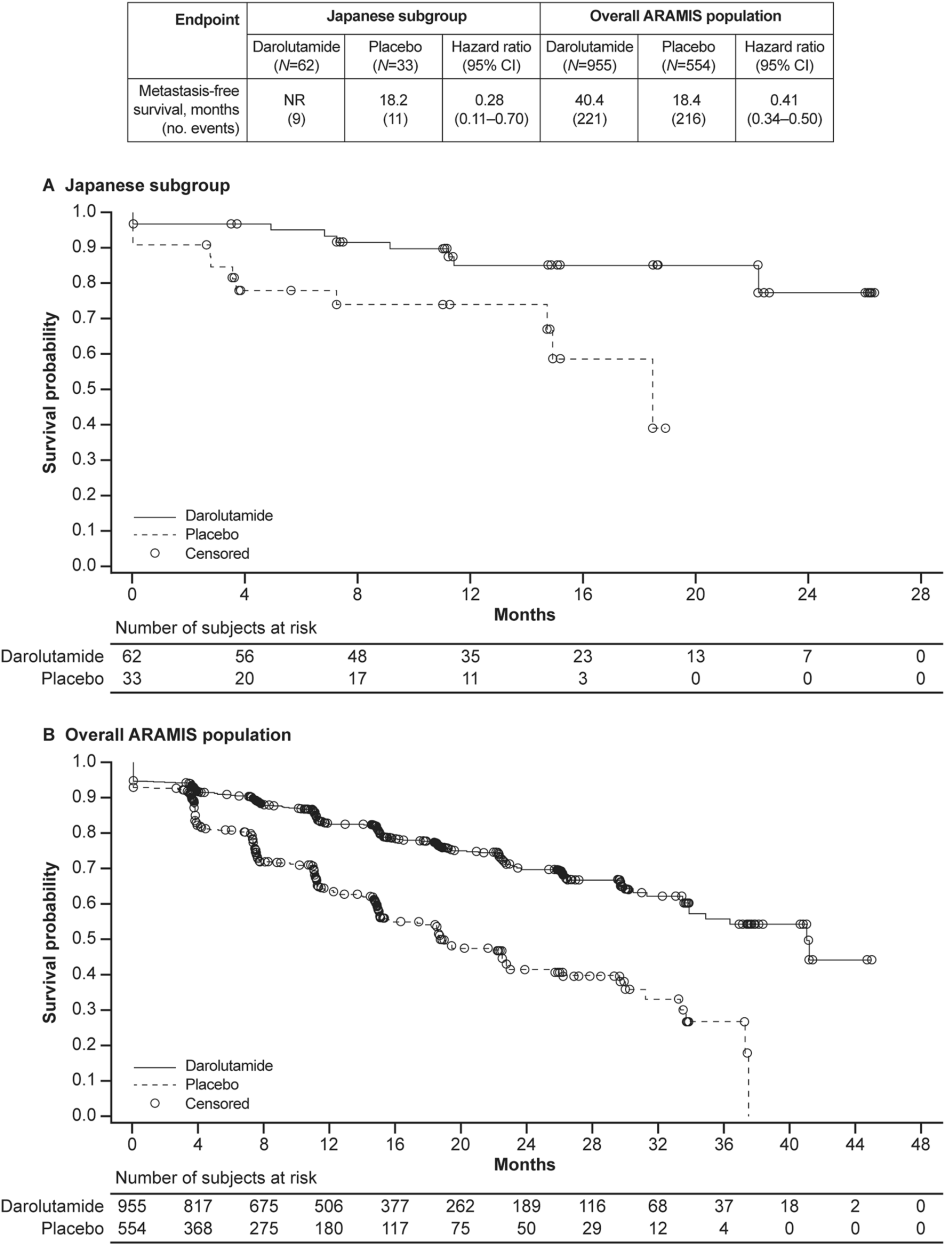
Metastasis-free survival in the Japanese subgroup (**a**) and overall ARAMIS population (**b**)

At the time of the primary analysis, 20 MFS events (9 in the darolutamide group, 11 in the placebo group) had been observed. MFS favored darolutamide (HR 0.28; 95% CI 0.11–0.70) in the Japanese subgroup. The median MFS was not reached with darolutamide vs. 18.2 months with placebo (Fig. 1).

At the cut-off date for the primary analysis, median OS was not reached due to the limited number of events in both groups but favored darolutamide in the Japanese subgroup patient population. After 23 pain progression events (13 in the darolutamide group, 10 in the placebo group) occurred, the median time to pain progression was not reached with darolutamide vs. 19.1 months with placebo. Median times to first cytotoxic chemotherapy and the first symptomatic skeletal event also were not reached in either treatment group (Table 2).

**Table 2:** Selected secondary and exploratory endpoints

Endpoint	Japanese subgroup			Overall ARAMIS population		
	Darolutamide (*N* = 62)	Placebo (*N* = 33)	Hazard ratio (95% CI)	Darolutamide (*N* = 955)	Placebo (*N* = 554)	Hazard ratio (95% CI)
Secondary endpoints						
Overall survival, months (no. events)	NR (3)	NR (2)	0.72 (0.12–4.31)	NR (78)	NR (58)	0.71 (0.50–0.99)
Time to pain progression, months (no. events)	NR (13)	19.1 (10)	0.52 (0.22–1.21)	40.3 (251)	25.4 (178)	0.65 (0.53–0.79)
Time to first cytotoxic chemotherapy, months (no. events)	NR (3)	NR (3)	0.46 (0.09–2.27)	NR (73)	38.2 (79)	0.43 (0.31–0.60)
Time to first SSE, months (no. events)	NR (1)	NR (2)	0.22 (0.02–2.48)	NR (16)	NR (18)	0.43 (0.22–0.84)
Exploratory endpoints						
Time to PSA progression, months (no. events)	NR (8)	7.4 (17)	0.13 (0.05–0.33)	33.2 (226)	7.3 (368)	0.13 (0.11–0.16)
Patients with PSA response ≥ 50%, n (%)	48 (77.4)	4 (12.1)	–	798 (83.6)	42 (7.6)	–
Patients with PSA response ≥ 90%, n (%)	27 (43.5)	0	–	486 (50.9)	10 (1.8)	–

*CI* confidence interval, *NR* not reached, *PSA* prostate-specific antigen, *SSE* symptomatic skeletal event

**Fig. 2:**
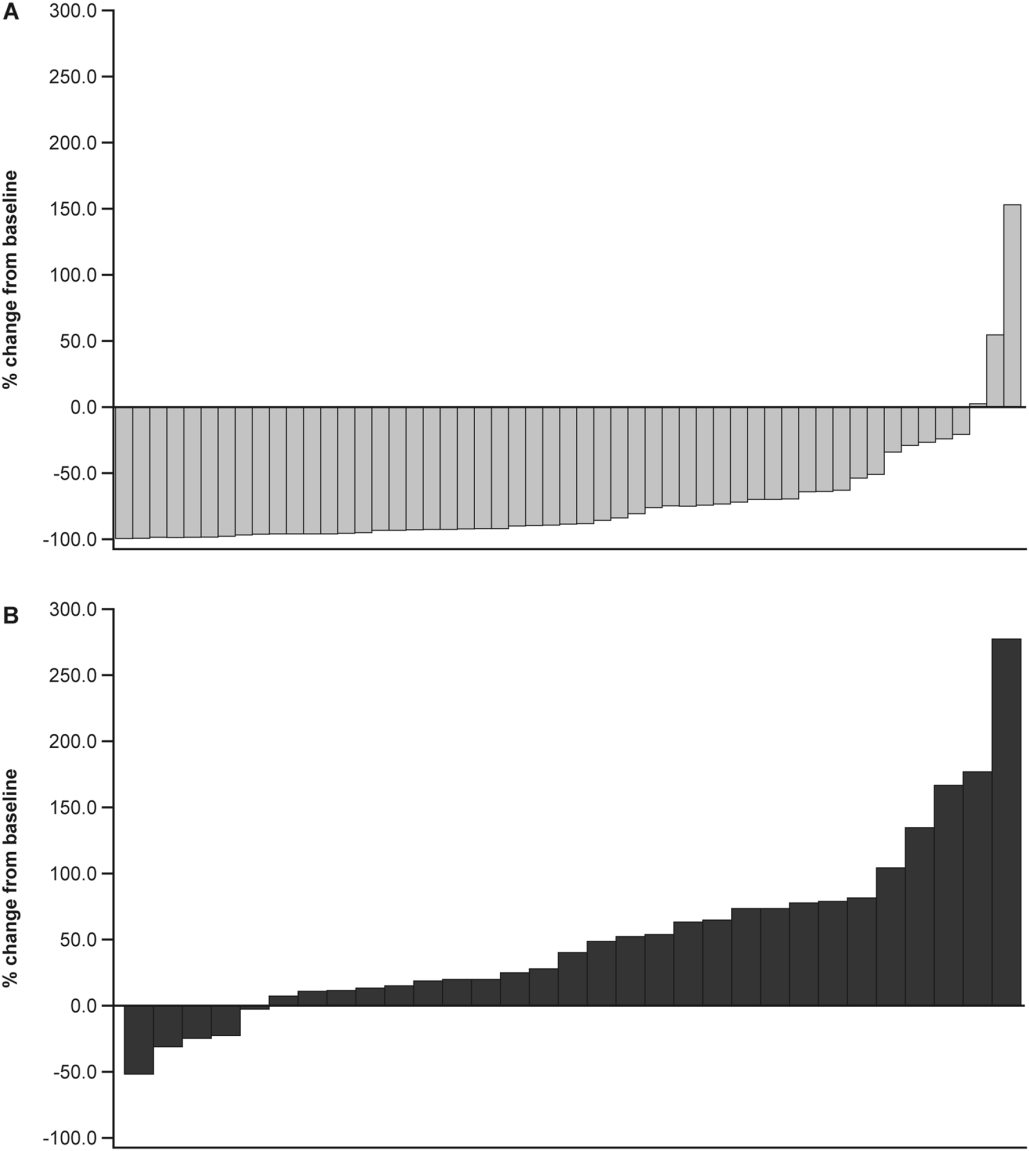
Waterfall plot of the percentage change in PSA from baseline at Week 16 in Japanese patients treated with darolutamide (**a**) and placebo (**b**). *PSA* prostate serum antigen. Nine patients in the darolutamide group and two patients in the placebo group had missing percentages. For patients who discontinued study treatment prior to Week 16, their PSA results prior to Week 16 were included and summarized

Analysis of time to PSA progression was performed after 25 PSA progression events (8 in the darolutamide group, 17 in the placebo group) occurred. The median time to PSA progression was not reached with darolutamide vs. 7.4 months with placebo (Table 2). A clear PSA response at 16 weeks was observed in Japanese patients (Fig. 2); 77.4% of patients with darolutamide vs. 12.1% of patients with placebo achieved a decrease in PSA level from baseline of ≥ 50%, while 43.5% of patients with darolutamide vs. zero patients with placebo attained a ≥ 90% decrease (Table 2).

## Safety

Overall, AEs were reported by 85.5 and 63.6% of patients receiving at least one dose of darolutamide and placebo, respectively, in the Japanese subgroup (Table 3). Most AEs were Grade 1–2 in severity (56.5% with darolutamide vs. 51.5% with placebo). In Japanese patients, any Grade 3 AE occurred in 25.8% of patients randomized to darolutamide and in 12.1% of those randomized to placebo; all events occurred in only one or two patients each and were not related to any particular organ system (Supplementary Table 3). The incidence of Grade 4 or 5 AEs was comparable between the two arms [two patients with darolutamide (3.2%; one Grade 4 hepatic function abnormality and one Grade 5 influenza) vs. one patient with placebo (3.0%; Grade 4 renal failure)]. Few Grade 3–5 AEs were considered by investigators to be drug-related [one Grade 3 neutropenia, one Grade 3 decreased neutrophil count, and one Grade 4 hepatic function abnormality with darolutamide (4.8%) vs. zero with placebo]. The incidence of serious AEs was higher in patients randomized to darolutamide than those assigned placebo (32.3 vs. 9.1%). TEAEs leading to permanent discontinuation of study drug occurred in 8.1% of patients with darolutamide and in 6.1% of patients with placebo in the Japanese subgroup (Table 3).

**Table 3:** Treatment-emergent adverse events overall and with ≥ 5% incidence globally

Patients with an adverse event, *n* (%)	Japanese subgroup				Overall ARAMIS population			
	Darolutamide (*N* = 62)		Placebo (*N* = 33)		Darolutamide (*N* = 954)		Placebo (*N* = 554)	
	Any Grade	EAIR per 100PY	Any Grade	EAIR per 100PY	Any Grade	EAIR per 100PY	Any Grade	EAIR per 100PY
Any	53 (85.5)	–	21 (63.6)	–	794 (83.2)	–	426 (76.9)	–
Serious	20 (32.3)	–	3 (9.1)	–	237 (24.8)	–	111 (20.0)	–
Discontinuation	5 (8.1)	–	2 (6.1)	–	85 (8.9)	–	48 (8.7)	–
Adverse events that occurred in ≥ 5% of patients in either group in the global patient population								
Fatigue	2 (3.2)	2.6	1 (3.0)	3.6	115 (12.1)	8.6	48 (8.7)	8.5
Back pain	5 (8.1)	6.5	1 (3.0)	3.6	84 (8.8)	6.3	50 (9.0)	8.8
Arthralgia	1 (1.6)	1.3	2 (6.1)	7.2	77 (8.1)	5.8	51 (9.2)	9.0
Diarrhea	3 (4.8)	3.9	4 (12.1)	14.4	66 (6.9)	4.9	31 (5.6)	5.5
Hypertension	2 (3.2)	2.6	1 (3.0)	3.6	63 (6.6)	4.7	29 (5.2)	5.1
Constipation	8 (12.9)	10.3	1 (3.0)	3.6	60 (6.3)	4.5	34 (6.1)	6.0
Pain in extremity	2 (3.2)	2.6	0	0	55 (5.8)	4.1	18 (3.2)	3.2
Anemia	3 (4.8)	3.9	0	0	53 (5.6)	4.0	25 (4.5)	4.4
Hot flush	1 (1.6)	1.3	0	0	50 (5.2)	3.7	23 (4.2)	4.1
Nausea	4 (6.5)	5.2	0	0	48 (5.0)	3.6	32 (5.8)	5.6
Urinary tract infection	1 (1.6)	1.3	1 (3.0)	3.6	47 (4.9)	3.5	28 (5.1)	4.9
Urinary retention	2 (3.2)	2.6	1 (3.0)	3.6	33 (3.5)	2.5	36 (6.5)	6.3

*PY* patient years

In the overall ARAMIS population, among the AEs that occurred in ≥ 5% of patients in either treatment group, constipation, reported as Grade 1–2 severity in all patients, was the only event reported in > 10% of darolutamide-treated patients in the Japanese subgroup (Table 3). With respect to the AEs of interest, bone fracture [seven patients with darolutamide (11.3%) vs. one patient with placebo (3.0%)] and fall [eight patients with darolutamide (12.9%) vs. one patient with placebo 3.0%)] demonstrated the most noticeable difference in incidence rates between darolutamide and placebo in Japanese patients (Table 4). Most fractures and falls were Grade 1 or 2, with only one Grade 3 fall (1.6%) and one Grade 3 fracture (1.6%); none led to dose modification or discontinuation and none were considered to be treatment related. Incidences of other AEs associated with androgen receptor inhibitors were generally comparable between darolutamide and placebo groups, accounting for variability due to small sample size. Seizures, mental impairment disorders, depressed mood disorders, and breast disorders/gynecomastia were not reported with darolutamide or placebo in Japanese patients.

**Table 4:** Treatment-emergent adverse events of interest

Patents with an adverse event, *n* (%)	Japanese subgroup				Overall ARAMIS population			
	Darolutamide (*N* = 62)		Placebo (*N* = 33)		Darolutamide (*N* = 954)		Placebo (*N* = 554)	
	Any Grade, *n *(%)	EAIR per 100PY	Any Grade *n* (%)	EAIR per 100PY	Any Grade, *n* (%)	EAIR per 100PY	Any Grade, *n* (%)	EAIR per 100PY
Bone fracture^a^	7 (11.3)	9.0	1 (3.0)	3.6	40 (4.2)	3.0	20 (3.6)	3.5
Fall	8 (12.9)	10.3	1 (3.0)	3.6	40 (4.2)	3.0	26 (4.7)	4.6
Fatigue/asthenic conditions^b^	4 (6.5)	5.2	1 (3.0)	3.6	151 (15.8)	11.3	63 (11.4)	11.1
Weight decrease	3 (4.8)	3.9	0	0	34 (3.6)	2.5	12 (2.2)	2.1
Seizures (any event)	0	0	0	0	2 (0.2)	0.1	1 (0.2)	0.2
Rash^c^	4 (6.5)	5.2	1 (3.0)	3.6	28 (2.9)	2.1	5 (0.9)	0.9
Cardiac disorders (SOC)	5 (8.1)	NA	1 (3.0)	NA	113 (11.8)	NA	41 (7.4)	NA
CNS vascular disorders^d^	2 (3.2)	2.5	0	0	16 (1.7)	1.2	10 (1.8)	1.7
Hypertension	2 (3.2)	2.6	1 (3.0)	3.6	70 (7.3)	5.2	33 (6.0)	5.8
Vasodilatation and flushing	1 (1.6)	1.3	0	0	54 (5.7)	4.0	23 (4.2)	4.1
Diabetes mellitus and hyperglycemia	1 (1.6)	1.3	0	0	22 (2.3)	1.6	12 (2.2)	2.1
Mental impairment disorders^e^	0	0	0	0	16 (1.7)	1.2	10 (1.8)	1.7
Depressed mood disorders^f^	0	0	0	0	17 (1.8)	1.3	8 (1.4)	1.4
Breast disorders/gynecomastia^g^	0	0	0	0	22 (2.3)	1.6	9 (1.6)	1.6

*CNS* central nervous system, *EAIR* exposure-adjusted incidence rate, *NA* not applicable, *PY* patient years, *SOC* standard of care

^a^Comprised of MedDRA preferred terms ‘any fractures and dislocations’, ‘limb fractures and dislocations’, ‘skull fractures’, ‘facial bone fractures and dislocations’, ‘spinal fractures and dislocations’, and ‘thoracic cage fractures and dislocations’

^b^Comprised of MedDRA preferred terms ‘asthenic conditions’, ‘disturbances in consciousness’, ‘decreased strength and energy’, ‘malaise’, ‘lethargy’, ‘asthenia’, and ‘fatigue’

^c^Comprised of MedDRA preferred terms ‘dermatitis’, ‘erythema’, ‘rash’, ‘rash macular’, ‘rash maculo-papular’, ‘rash papular’ and ‘rash pustular’

^d^Comprised of MedDRA preferred terms ‘cerebral infarction’, ‘cerebral ischemia’, ‘cerebrovascular accident’, ‘ischemic stroke’, and ‘transient ischemic attack’

^e^Comprised of MedDRA preferred terms ‘memory impairment’ and ‘cognitive disorder’

^f^Comprised of MedDRA preferred terms ‘depression’ and ‘depressed mood’

^g^Comprised of MedDRA preferred terms ‘gynecomastia’, ‘breast discomfort’, ‘breast induration’, ‘breast pain’, ‘breast tenderness’

## Discussion

In this pre-specified analysis of Japanese patients with nmCRPC in the phase III ARAMIS study, efficacy and safety of darolutamide were generally consistent with the overall ARAMIS population. Differences were noted in baseline and clinical characteristics between the overall ARAMIS population and the small number of patients in the Japanese subgroup, some of which may be related to differences in clinical practice. In contrast to Western clinical practice guidelines, treatment algorithms in Japan include ADT for localized prostate cancer in patients for whom prostatectomy or radiation is not indicated [37, 38]. This may explain the greater number of prior hormonal therapies received by the Japanese subgroup of ARAMIS compared with the overall study population. The Japanese subgroup also included greater proportions of older patients and patients with a higher Gleason score (≥ 7) and/or more advanced primary tumor classification (T3a), in line with other studies of Japanese or Asian patients with nmCRPC [39, 40].

Efficacy outcomes of darolutamide across the small number of patients enrolled and survival events that occurred in the Japanese subgroup were generally comparable with those of the overall ARAMIS population. Darolutamide prolonged MFS in the Japanese subgroup, with a HR of 0.28 (95% CI 0.11–0.70). Although median OS was not reached in the Japanese subgroup, it trended toward favoring darolutamide, with a HR of 0.72 (95% CI 0.12–4.31). Other secondary and exploratory endpoints, including the time to pain progression, time to PSA progression, and PSA response, also favored darolutamide, providing further evidence to support its clinical benefit in Japanese men with nmCRPC.

Darolutamide was well tolerated, with ≥ 93% of patients receiving ≥ 90% of the planned dose in both the Japanese subgroup and the overall ARAMIS population. Safety outcomes of darolutamide in the Japanese population were generally consistent with those of the overall ARAMIS population; similar incidences of any grade AE, Grade 1–2, and Grade 3–4 events, and TEAEs leading to permanent discontinuation of study drug were reported among darolutamide-treated patients. While reports of Grade 5 AEs were approximately 59% lower in the Japanese subgroup compared with those in the overall ARAMIS population, serious AEs occurred more frequently in darolutamide-treated patients in the Japanese subgroup (32.3%) than in the overall ARAMIS population (24.8%). However, the correlation with active study drug was low in the majority of these cases.

Certain differences were noted between Japanese and overall ARAMIS populations with respect to the AEs most frequently reported with darolutamide. Fatigue, which occurred more commonly with darolutamide than placebo in the overall ARAMIS population [26], was similar between treatment arms in the Japanese subgroup. Also, incidences of constipation, falls, and fractures tended to be higher with darolutamide than placebo in the Japanese subgroup but showed no difference between treatment groups in the overall ARAMIS population at primary analysis.

As no remarkable differences in darolutamide pharmacokinetic parameters exist between Japanese and Western patients [35], this is unlikely to account for any potential differences in the safety profile of darolutamide observed in the Japanese and overall ARAMIS populations. The higher incidence of fractures associated with darolutamide in the Japanese subgroup may be related to more elderly patients (aged ≥ 85 years) in the darolutamide group as compared with the placebo group (19.4 vs. 6.1%), since advanced age is a significant risk factor for fracture in men with prostate cancer [41, 42]. In addition, the longer duration of prior ADT in the darolutamide group may have contributed to the higher incidence of fractures, consistent with previous studies demonstrating an association between duration of ADT and fracture risk in both Western and Japanese [41, 43–46]. As ADT-related fracture risk also may increase with advancing age [47], an additive effect may have been induced by the higher proportion of patients aged ≥ 85 years combined with greater exposure to ADT in the darolutamide group. The difference in age distribution between the treatment arms in the Japanese subgroup also may have contributed to the higher incidence of constipation with darolutamide vs. placebo [48–50]. Considered together, the small sample size, small number of AEs observed overall, and differences in duration of exposure to study treatment (14.8 months with darolutamide vs. 10.9 months with placebo), these potential confounding variables suggest that differences in safety between darolutamide and placebo in the Japanese subgroup should be interpreted with caution and further investigations should be considered.

The safety observations described herein may be best understood in the context of tumor extension, age, and chronic comorbidities in the Japanese subgroup. The darolutamide group enrolled more elderly patients and patients with locally advanced cancer compared with the placebo group. The overall median age of the Japanese subgroup was higher than that of the global population, and similarly to the global population, almost all patients had at least one comorbidity. In line with our findings, subgroup analyses of efficacy in Japanese or Asian patients with nmCRPC treated with apalutamide in the phase III SPARTAN trial found the delay in the development of metastases to be consistent with the overall ARAMIS population. In general, safety profiles also aligned with those of the overall trial populations, with few differences such as increased incidence of rash [39], possibly related to ethnicity. Data from Asian patients with nmCRPC in PROSPER have not been published to date. In subgroup analyses of Asian patients with mCRPC treated with enzalutamide in the phase III PREVAIL trial, AEs commonly reported by patients treated with enzalutamide were generally similar to those reported in the overall study population [51, 52] Although a small number of AEs including falls occurred more frequently in the subgroup, the authors highlight that any differences should be interpreted with caution due to the small patient numbers.

In conclusion, improvements in MFS and other efficacy endpoints observed with darolutamide in the Japanese subgroup are generally consistent with the overall ARAMIS population, supporting the clinical benefit of darolutamide in Japanese patients with nmCRPC. Darolutamide was well tolerated in Japanese patients. Given the small number of patients in the Japanese subgroup, it is impossible to conclude with certainty whether differences in the safety profile exist between Japanese and overall ARAMIS populations.

## Electronic supplementary material

Below is the link to the electronic supplementary material.

Supplementary material 1 (DOCX 24 kb)
