# Supplementary material for: Efficacy and safety of darolutamide in Japanese patients with nonmetastatic castration-resistant prostate cancer: a sub-group analysis of the phase III ARAMIS trial
Source: Int J Clin Oncol. 2020 Nov 23;26(3):578–90. doi: 10.1007/s10147-020-01824-5 (PMC7895789; doi:10.1007/s10147-020-01824-5)
Supplement: Supplementary file 1 — Supplementary material 1 (DOCX 24 kb) [file 10147_2020_1824_MOESM1_ESM.docx]

# Data Supplement

## Supplementary Table 1. Treatment duration

|  | Japanese subgroup | | Overall ARAMIS population | |
| --- | --- | --- | --- | --- |
| Treatment duration | **Darolutamide  (*N* = 62)** | **Placebo  (*N* = 33)** | **Darolutamide  (*N* = 954)** | **Placebo  (*N* = 554)** |
| Median (range), months  ≤ 1 month, n (%)  > 1 to ≤ 6 months, n (%)  > 6 to ≤ 12 months, n (%)  > 12 to ≤ 18 months, n (%)  > 18 to ≤ 24 months, n (%)  > 24 to ≤ 30 months, n (%)  > 30 to ≤ 36 months, n (%)  > 36 months, n (%) | 14.8 (0.4–26.1)  1 (1.6)  5 (8.1)  17 (27.4)  16 (25.8)  16 (25.8)  7 (11.3)  0  0 | 10.9 (2.1–18.6)  0  9 (27.3)  12 (36.4)  9 (27.3)  3 (9.1)  0  0  0 | 14.8 (0–44.3)  16 (1.7)  78 (8.2)  280 (29.4)  172 (18.0)  188 (19.7)  133 (13.9)  46 (4.8)  41 (4.3) | 11.0 (0.1–40.5)  9 (1.6)  136 (24.5)  187 (33.8)  93 (16.8)  67 (12.1)  44 (7.9)  9 (1.6)  9 (1.6) |

## Supplementary Table 2. Dosages

|  | Japanese subgroup | | Overall ARAMIS population | |
| --- | --- | --- | --- | --- |
| Percent of planned dose | **Darolutamide  (*N* = 62)** | **Placebo  (*N* = 33)** | **Darolutamide  (*N* = 954)** | **Placebo  (*N* = 554)** |
| Median (SD) | 100.0 (9.5) | 100.0 (7.4) | 100.0 (5.4) | 100.0 (4.1) |
| > 30% to 60%, N (%) | 2 (3.2) | 1 (3.0) | 7 (0.7) | 3 (0.5) |
| > 60% to 90%, N (%) | 2 (3.2) | 1 (3.0) | 20 (2.1) | 6 (1.1) |
| > 90% to 100%, N (%) | 58 (93.5) | 31 (93.9) | 927 (97.2) | 545 (98.4) |

*SD* standard deviation

## Supplementary Table 3. Grade 3 treatment-emergent adverse events in Japanese patients

| Patients with an adverse event, n (%) | Darolutamide  (*N* = 62) | Placebo  (*N* = 33) |
| --- | --- | --- |
| Treatment-emergent adverse events | | |
| Any | 16 (25.8) | 4 (12.1) |
| Bladder neoplasm | 2 (3.2) | 0 |
| Hydronephrosis | 2 (3.2) | 0 |
| Abscess | 1 (1.6) | 0 |
| Alanine aminotransferase increased | 1 (1.6) | 0 |
| Anemia | 1 (1.6) | 0 |
| Angina pectoris | 1 (1.6) | 0 |
| Arrhythmia | 1 (1.6) | 0 |
| Aspartate aminotransferase increased | 1 (1.6) | 0 |
| Asthma | 1 (1.6) | 0 |
| Bronchitis | 1 (1.6) | 0 |
| Cataract | 1 (1.6) | 0 |
| Colon cancer | 1 (1.6) | 0 |
| Decreased appetite | 1 (1.6) | 1 (3.0) |
| Fall | 1 (1.6) | 0 |
| Fracture | 1 (1.6) | 0 |
| Gingivitis | 1 (1.6) | 0 |
| Hematuria | 1 (1.6) | 0 |
| Iron deficiency anemia | 1 (1.6) | 0 |
| Neutropenia | 1 (1.6) | 0 |
| Neutrophil count decreased | 1 (1.6) | 0 |
| Pancreatic carcinoma | 1 (1.6) | 0 |
| Pneumonia | 1 (1.6) | 0 |
| Postoperative ileus | 1 (1.6) | 0 |
| Pulmonary mass | 1 (1.6) | 0 |
| Rectal cancer | 1 (1.6) | 1 (3.0) |
| Urinary retention | 1 (1.6) | 0 |
| Diarrhea | 0 | 1 (3.0) |
| Fatigue | 0 | 1 (3.0) |
| Gastric cancer | 0 | 1 (3.0) |
| Urinary tract infection | 0 | 1 (3.0) |
